# Regional-Scale Migrations and Habitat Use of Juvenile Lemon Sharks (*Negaprion brevirostris*) in the US South Atlantic

**DOI:** 10.1371/journal.pone.0088470

**Published:** 2014-02-26

**Authors:** Eric A. Reyier, Bryan R. Franks, Demian D. Chapman, Douglas M. Scheidt, Eric D. Stolen, Samuel H. Gruber

**Affiliations:** 1 Kennedy Space Center Ecological Program and InoMedic Health Applications, Kennedy Space Center, Florida, United States of America; 2 Department of Biology, Florida Southern College, Lakeland, Florida, United States of America; 3 Institute for Ocean Conservation Science, Stony Brook University, Stony Brook, New York, United States of America; 4 Bimini Biological Field Station, Bimini, Bahamas; School of Environment & Life Sciences, University of Salford, United Kingdom

## Abstract

Resolving the geographic extent and timing of coastal shark migrations, as well as their environmental cues, is essential for refining shark management strategies in anticipation of increasing anthropogenic stressors to coastal ecosystems. We employed a regional-scale passive acoustic telemetry array encompassing 300 km of the east Florida coast to assess what factors influence site fidelity of juvenile lemon sharks (*Negaprion brevirostris*) to an exposed coastal nursery at Cape Canaveral, and to document the timing and rate of their seasonal migrations. Movements of 54 juvenile lemon sharks were monitored for three years with individuals tracked for up to 751 days. While most sharks demonstrated site fidelity to the Cape Canaveral region December through February under typical winter water temperatures, historically extreme declines in ocean temperature were accompanied by rapid and often temporary, southward displacements of up to 190 km along the Florida east coast. From late February through April each year, most sharks initiated a northward migration at speeds of up to 64 km day^−1^ with several individuals then detected in compatible estuarine telemetry arrays in Georgia and South Carolina up to 472 km from release locations. Nineteen sharks returned for a second or even third consecutive winter, thus demonstrating strong seasonal philopatry to the Cape Canaveral region. The long distance movements and habitat associations of immature lemon sharks along the US southeast coast contrast sharply with the natal site fidelity observed in this species at other sites in the western Atlantic Ocean. These findings validate the existing multi-state management strategies now in place. Results also affirm the value of collaborative passive arrays for resolving seasonal movements and habitat preferences of migratory coastal shark species not easily studied with other tagging techniques.

## Introduction

It is now widely recognized that as a group, sharks are unusually susceptible to overfishing, relative to most other marine fishes, due to their slow growth, late age of maturation, and low fecundity [1], [2]. However, management of shark stocks is further complicated by a growing realization that many species undertake seasonal migrations spanning hundreds or thousands of kilometers in which they transit through jurisdictions with incongruous fishing regulations and enforcement strategies [3], [4]. Prudent management in a given area can be largely negated by unsustainable harvest or habitat degradation in other portions of a species range. Better understanding the geographic scale, directionality, and timing of shark migrations will help guide shark conservation efforts in coming decades as oceans are further stressed by habitat loss and ever-growing human dependence on marine resources. Specifically, migration data can be used to resolve stock boundaries, refine fishing seasons and catch quotas, limit shark bycatch, identify high value habitats (such as Habitat Areas of Particular Concern in US waters), and establish time-area closures or marine reserves [5].

The migrations of coastal shark species are often closely coupled with seasonal variations in water temperature [6]–[8]. These migrations appear to be adaptations to stay within a preferred temperature range, exploit seasonally productive foraging grounds, utilize optimal mating and parturition sites, or a combination thereof. Along the US Atlantic and Gulf coasts, fishery landings and field surveys demonstrate that most coastal sharks become more abundant in northern and inshore portions of their range as waters warm in spring [9]–[15]. Females use nearshore waters and estuaries as pupping grounds where neonates remain through summer, presumably taking advantage of high prey availability and reduced predation [16]. By fall, individuals again shift southward and/or offshore. Yet even in this region where shark behavior has been a priority research focus for several decades, migrations have not been resolved in detail for most species due to the difficulties of following individual animals as they travel long distances through open water.

Passive acoustic telemetry is steadily gaining favor as an approach for resolving the detailed movements of fishes, including sharks, in estuarine and coastal settings [17]. Passive telemetry utilizes an array of submerged acoustic receivers deployed to autonomously record the presence of fish carrying acoustic transmitters. Individual animals can therefore be tracked for intervals much longer than is possible with manual telemetry where movements are recorded with a mobile (usually boat-based) receiver. One limitation, however, is that detections are only obtained when animals pass within a few hundred meters of a receiver. Consequently, a large percentage of passive telemetry studies of sharks to date [18]–[24], have occurred at insular locations or targeted reef-associated species where site fidelity is expected to be high. Studies of migratory shark species in continental settings [25]–[28] are often more challenging and generally yield data on individual animals for days to months, and encompass small sections of coastline. Theoretically, however, passive arrays are readily up-scalable so as to be suitable for resolving multi-year, regional-scale migrations and habitat associations in the coastal realm. Such efforts are arguably of greater management value since they better identify natural and anthropogenic risks facing long-lived marine species including sharks.

The life history of the lemon shark (*Negaprion brevirostris*) has received considerable scrutiny compared with most coastal sharks. Not only is it widely distributed throughout the western Atlantic from North Carolina to Brazil, the Gulf of Mexico, Caribbean Sea, and tropical eastern Atlantic and eastern Pacific, it is an apex predator in several habitats including turbid estuaries, seagrass beds, mangroves, and coral reefs [29], [30]. Moreover, the lemon shark exhibits life history traits that leave it prone to overfishing. They grow slowly, only reaching sexual maturity when 225–240 cm total length and 11–13 years of age [31]. Fecundity is also low with females producing only 4–18 offspring every other year [32]. Like many large sharks, the species has been heavily fished throughout its range, is currently listed by the IUCN as a near-threatened species, and is the subject of growing management concern.

Studies of the lemon shark using mark-recapture, acoustic telemetry, and genetic techniques in the Bahamas [33]–[37], south Florida [38], Caribbean [39], and Brazil [24], [40], [41] demonstrate that juveniles maintain fidelity to their natal nurseries for several years, have home ranges that expand gradually with age, and show little tendency for long distance dispersal until they approach adulthood. However, recent findings from the US southeast coast suggest a very different strategy with young lemon sharks forming high density aggregations each winter in the surf zone at Cape Canaveral, Florida, with evidence of a northward spring migration as far as North Carolina [42]. Adult lemon sharks in the region exhibit a similar migratory behavior but with winter aggregations occurring near Jupiter, Florida [43], 170 km south of Cape Canaveral. We argue here that better understanding details of these aggregations and migration patterns is necessary to guide long-term management of the species in the US South Atlantic region. Therefore, the specific objectives of this study were to: (1) use a collaborative regional-scale passive acoustic array to resolve the degree of site fidelity of juvenile lemon sharks to Cape Canaveral, and (2) document the timing, rate, destinations, and temperatures associated with their seasonal migrations.

## Materials and Methods

### Ethics Statement

Lemon shark collection and handling was performed in accordance with a State of Florida Special Activity License (permit SAL-09-512-S) and the study was specifically approved by the Kennedy Space Center Institutional Animal Care & Use Committee (permit GRD-06-049).

### Study Area

**Figure 1:**
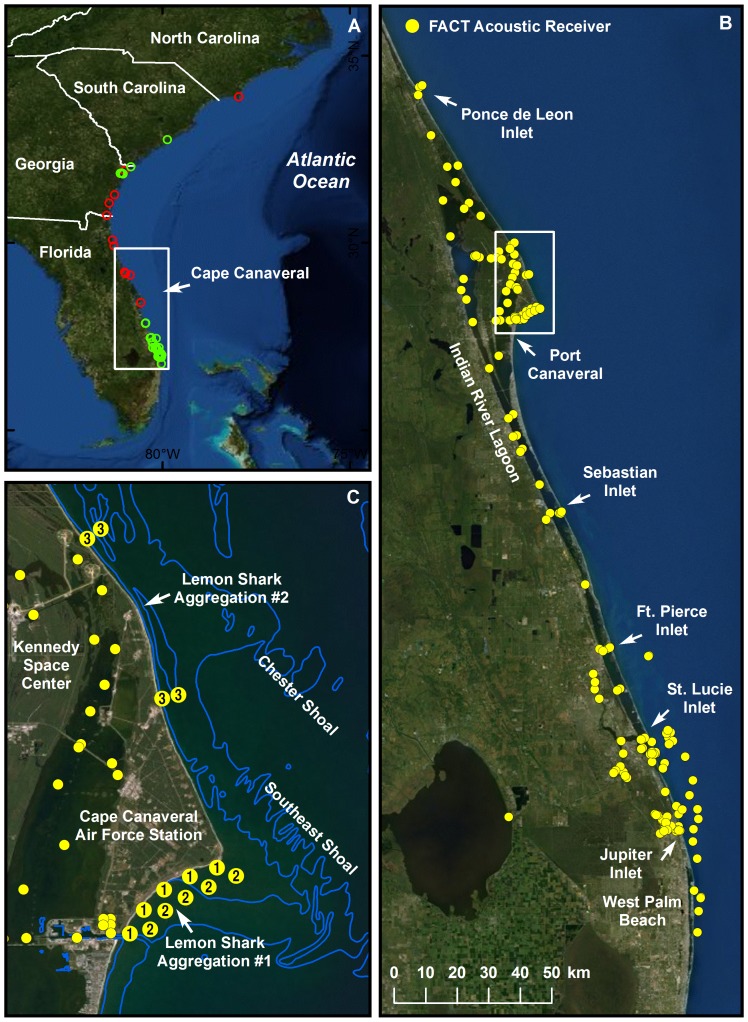
Passive acoustic tracking of lemon sharks in the US South Atlantic region. **A)** Overall study region including locations of all lemon shark acoustic detections (green circles) and historic angler recaptures (red circles) from sharks released at Cape Canaveral. B) Map of the full FACT Array including all passive acoustic receivers (yellow dots). C) Close-up of the Canaveral Array including locations of two important lemon shark aggregation sites. Nearshore receivers are numbered 1–3 which correspond to the year of the study they were deployed.

Tagging of juvenile lemon sharks was conducted at Cape Canaveral, east-central Florida (28.5° N, Fig. 1) from the beaches of Cape Canaveral Air Force Station and NASA’s Kennedy Space Center. The shoreline here is among the most pristine of the Florida Atlantic coast with no residential or commercial development. Habitat disturbance is limited to space launch infrastructure set back from the beach several hundred meters. Due to security concerns associated with launch activities, public beach access has been prohibited along 45 km of this coast since the mid-1950s although vessel-based activities (including fishing) are permitted. Nearshore waters are characterized by the expansive Southeast and Chester Shoals (minimum depth 1–3 m), with adjacent waters reaching 15 m. Bottom sediments are a mosaic of sand, shell, and mud with little hard-bottom substrate near the beach [44]. The shoreline exhibits longshore troughs that are partially sheltered from the surf zone by parallel sandbars. Juvenile lemon sharks up to 2 m long commonly aggregate within these troughs [42]. The Indian River Lagoon system lies directly inland of the study site, however the nearest ocean inlets are Ponce de Leon Inlet (60 km north) and Sebastian Inlet (62 km south) as well as a small lock system in nearby Port Canaveral. Salinity remains roughly 35 psu year-round and tides have an amplitude of ∼ 1 m. The Canaveral region is a recognized climatic transition zone between warm-temperate and sub-tropical biogeographic realms [45]. Winter water temperatures remain above 15°C most years, however periodic cold fronts can induce brief but rapid declines in coastal water temperature.

### Shark Tagging

A total of 54 juvenile lemon sharks were collected from two recurring aggregation sites at Cape Canaveral (Fig. 1) over three successive fall-winter periods from 2008 to 2010. The number of sharks using each site occasionally exceeds several hundred individuals. All animals were collected from shore using a 3.7 m radius monofilament cast net. After capture, sharks were transferred to a 125-liter tank where they were placed ventral side up. The inverted position induced tonic immobility, after which a 25 mm incision was made parallel to the ventral midline and anterior to the cloaca. A coded acoustic transmitter was inserted into the peritoneal cavity and the incision was then closed with 2–4 absorbable sutures (Look™ Polysyn) and cyanoacrylate adhesive (Vetbond™, 3 M Corporation). In the first year, all sharks were fitted with Vemco V9-2H tags (5 g in air, 180 sec. nominal delay, ∼270 day battery life). In subsequent years, larger Vemco V16-6H tags (34 g in air, 90 sec. nominal delay, 6.4 year battery life) were used. Sharks were also marked with external dart tags offering a reward in case of angler recapture and then released on site. Total time from capture to release was usually 10–15 minutes.

### Florida Atlantic Coast Telemetry (FACT) Array

Movements of tagged sharks were monitored via the Florida Atlantic Coast Telemetry (FACT) Array, a regional-scale passive acoustic array maintained by several marine research organizations. During this study, the FACT Array consisted of 160–180 acoustic receivers (Vemco VR2 and VR2W) deployed over 300 km of the Florida east coast from West Palm Beach (26.5° N) to Ponce de Leon Inlet (29.1° N; Fig. 1). FACT monitored multiple habitats including beaches and nearshore reefs/wrecks in the open Atlantic Ocean as well as estuarine waters of the adjacent Indian River Lagoon. Special attention was taken to anchor receivers at migratory chokepoints including all ocean inlets as well as natural constrictions, causeway channels, and river mouths. In addition to FACT, several other compatible passive acoustic arrays were deployed in the US South Atlantic. Most notably, an expansive array was established in estuarine and riverine waters of Georgia, South Carolina, and North Carolina by January 2011, during the third year of this study. Arrays were also located at various locations in the Florida Keys, Bahamas, and Chesapeake Bay for the duration of this study.

At Cape Canaveral, the number of FACT receivers (referred to herein as the Canaveral Array) was expanded each winter (Fig. 1). In December 2008, five “nearshore” receivers were deployed 250 m off the beach at a large lemon shark aggregation site south of Cape Canaveral. In December 2009, an additional “offshore” row of five receivers was installed 1250 m from the beach at this same site. Finally, in December 2010, four additional receivers were added just north of Cape Canaveral near a second aggregation site, bringing the total local receiver count to 14. Mean depth of nearshore and offshore stations were 3.7 and 6.7 m, respectively. All receivers were bracketed to large sand screws and downloaded using SCUBA at six-month intervals.

Daily water temperature (°C) and wave height (m) within the Canaveral Array was obtained from NOAA buoy #41113 moored 5 km east of Port Canaveral. Water temperature was also measured using temperature recorders (HOBO™ loggers, Onset Corporation) attached to receivers at Ponce de Leon Inlet, St. Lucie Inlet, and Jupiter Inlet. When sharks were detected at nearshore locations lacking loggers, surface water temperature was estimated using NOAA AVHRR satellite imagery (available at http://marine.rutgers.edu/mrs/sat_data). Air temperature data from 1901–2011, used to provide historic context as to the relative severity of winter temperatures experienced at Cape Canaveral during the study, was obtained from the nearby Titusville National Climatic Data Center Station #088942. The relationship between air and water temperature at Cape Canaveral was explored using Spearman’s rank correlation for all 984 days of the study when both values were available.

### Acoustic Array Performance

Assessing the performance of Canaveral Array receivers over a broad spectrum of ocean conditions was important given that lemon sharks frequent the surf zone where wave action may hinder transmitter detection. The large study area made it impractical to quantify detection distances throughout the entire array. We instead deployed a range-test transmitter with a 3-min fixed interval at a single location for 162 continuous days to gauge detection rates in relation to changing habitat conditions. This transmitter, which had a signal strength (160 dB) identical to that used in most sharks, was deployed on a small rod midway between a nearshore and offshore station (depth 4.2 and 8.5 m, respectively). The transmitter was thus 750 m from the shore and 500 m away from each receiver. We tested daily detection probability of this transmitter as a function of water depth (shallow vs. deep receiver), daily wave height, and daily water temperature, using a limited set of nested generalized least squares models [46] within the nlme package [47] of R. To account for potential serial autocorrelation between successive days, we investigated models incorporating simple autoregressive correlation structures ARMA and AR1. Because variance in daily detection rate appeared to differ between depths, we also considered models which allowed for this difference in the variance structure. Once we had chosen the best correlation and variance structure, we used model selection based on adjusted Akaike Information Criteria (AIC_c_) [48] to compare the full model with both interaction terms to all simpler models (i.e., one or more terms removed).

### Shark Habitat Use and Movement Analyses

Analyses of shark movements were constrained to data collected from December 2008 through December 2011 (37 months). To avoid inclusion of false detections resulting from code collisions and background noise, detections at a receiver were deemed valid only if two or more occurred within a 30-min period for a given shark unless detections for that individual were also recorded at a receiver < 5 km away on the same date. A scatterplot was created to graphically depict individual lemon shark position along six pre-defined regions of the SE US coastline including: (1) coastal waters at Cape Canaveral (14 FACT stations), (2) southeast Florida from Sebastian Inlet to West Palm Beach (∼50 FACT stations), (3) Ponce de Leon Inlet (4 FACT stations), (4) estuarine waters of the Indian River Lagoon (∼110 FACT stations), as well as in passive arrays in (5) Georgia, and (6) South Carolina.

Traditional measures of animal home range size (e.g., kernel density estimates) derived from passive receivers in the open ocean are likely to be misleading. We instead sought to identify individual-based and environmental variables that helped predict lemon shark presence at Cape Canaveral by developing a series of 72 *a priori* candidate logistic regression-type generalized linear models [49].The support for each “residency model” was measured by its AIC_c_ value [48]. Our binomial response variable was the daily presence/absence of an individual shark anywhere within the Canaveral Array (not detections at specific receivers). Individual-based explanatory variables considered were shark sex, log-transformed size at capture, size class (large vs. small), and days at liberty. We also considered days at liberty as a categorical variable with four levels to explore the scale of this effect on shark detection probability. Environmental variables considered included water temperature (°C), the magnitude of water temperature change over the previous 3, 7, 14, and 30-day intervals (termed Δ3temp, Δ7temp, Δ14temp, Δ30temp), day length (hours), wave height (m), and month of year. Water temperature and day length were highly correlated and thus never included in the same model. Individual sharks were considered a random effect to account for any individual heterogeneity. Study Year was included as a random effect since the expanding array footprint each winter resulted in growing detection probability through time. Month crossed with year was considered a random effect to account for temporal patterns not explained by any fixed effects. Sharks present at Cape Canaveral for less than one week (n = 5) provided limited information and were not included.

To account for potential serial autocorrelation in daily detection probability, we included state dependence and time series approaches [50]. Specifically, we created six state dependence variables which coded for whether or not an individual shark was detected at Cape Canaveral over the previous 1–6 days. We then considered six state dependence models which included the first order through sixth order autocorrelation terms added to the full model (e.g., 1 day lag + 2 day lag). We used AIC_c_ to decide which state dependence model had the best support. We also considered time series models which incorporated the serial autocorrelation structure directly into the generalized linear mixed effects models, using function glmmPQL from the MASS package in R version 2.14.1 [51]. Because these models were fit using quasi-likelihood methods, we could not use this formulation directly in model selection; instead they were used to evaluate the use of state dependence variables to address the serial autocorrelation. Once we decided on the optimal random effects and state dependence structure, we fit all 72 candidate residency models with this structure using the lme4 package [52] in R version 2.14.1 [51].

In addition to residency, we examined depth preferences of lemon sharks in the Canaveral Array by comparing the distribution of detections on the nearshore receiver row vs. offshore receiver row using a χ^2^ test. Further, to explore whether shark detections varied across the day as a result of onshore-offshore movements, time of each detection was rounded to the nearest hour and the resulting distribution was also explored using a χ^2^ test with the null hypothesis being equal detections throughout a diel cycle. Only data collected after November 2009, after which equal numbers of receivers were deployed in each row, were included.

To provide a range for lemon shark migration speeds along the coastline, rate of movement was calculated for all occasions when sharks transitioned between our six pre-defined coastal regions (e.g., Cape Canaveral, Ponce Inlet, SE Florida). These movements exceeded 50 km in all instances. Rates were noted as km day^−1^, and in body lengths sec^−1^ for events which occurred within six months of shark tagging. Distance was measured as the straight-line distance through water between receivers. We considered movements from Cape Canaveral to either Ponce de Leon Inlet (north) or the Sebastian Inlet-West Palm Beach region (south) as providing truest estimates of migration rates. These migrations follow a relatively linear coastline and all tidal inlets were monitored with acoustic receivers, allowing us to account for any excursions into the Indian River Lagoon. Differences in swimming speed between direction (north vs. south) and sex were compared using Student’s t-tests.

## Results

A total of 54 juvenile lemon sharks (27 males, 27 females) were tagged, most during one of three successive fall-winter seasons: December 2008 (n = 9), December 2009-January 2010 (n = 23), and November 2010-March 2011 (n = 20) although transmitters returned by anglers were implanted in new sharks during June 2010 (n = 1) and April 2011 (n = 1; Table 1). Captured sharks ranged in size from 610 to 1430 mm fork length (FL) with a mean of 840 mm FL. Shark size was similar across years (ANOVA, F_2,51_ = 0.84, *P* = 0.44) and between sexes (Wilcoxon Rank Sum Test, W = 376.5, *P* = 0.84).

**Table 1:** Summary information for all 54 lemon sharks tagged at Cape Canaveral.

	Year of Release			
	Winter 2008–2009	Winter 2009–2010	Winter 2010–2011	Total
Sharks Released	9	23	22	54
Sex Ratio (F:M)	5:4	11:12	11:11	27:27
Size (mm fork length; mean ± SD)	867±143	776±162	896±211	840±186
Days at Liberty (mean ± SD)	148±141	236±300	224±156	217±226
Position Detections (mean ± SD)	596±715	1022±1838	591±721	775±1316
Receiver Stations Visited	4.0±1.1	11.3±6.2	9.0±4.3	9.1±5.5
Max. Observed Displacement (km)				
North (mean ± SD)	47±43	29±39	131±156	74±114
South (mean ± SD)	2±1	107±80	24±43	56±74

Days at liberty equals the number of days between release and last detection. Maximum displacement means the farthest known detection north and south of release point.

### Acoustic Array Performance

The performance trial of the Canaveral Array ran for 162 days with an overall daily detection rate of a range test transmitter deployed near the surf zone being 64.2% from a distance of 500 meters. Performance varied markedly through time with daily detection rates ranging from 0.4–95.3%. The best supported model had main effects for both wave height and temperature (P <0.001; see Table S1 for model details). As wave height increased, tag detection rates decreased, and as water temperature increased, tag detection rates increased. Water depth was not a significant factor in this setting with the nearshore (4.2 m deep) and offshore (8.5 m deep) receivers performing similarly with daily detection rates of 64.5% and 63.9%, respectively.

### Shark Residency and Habitat Use at Cape Canaveral

**Figure 2:**
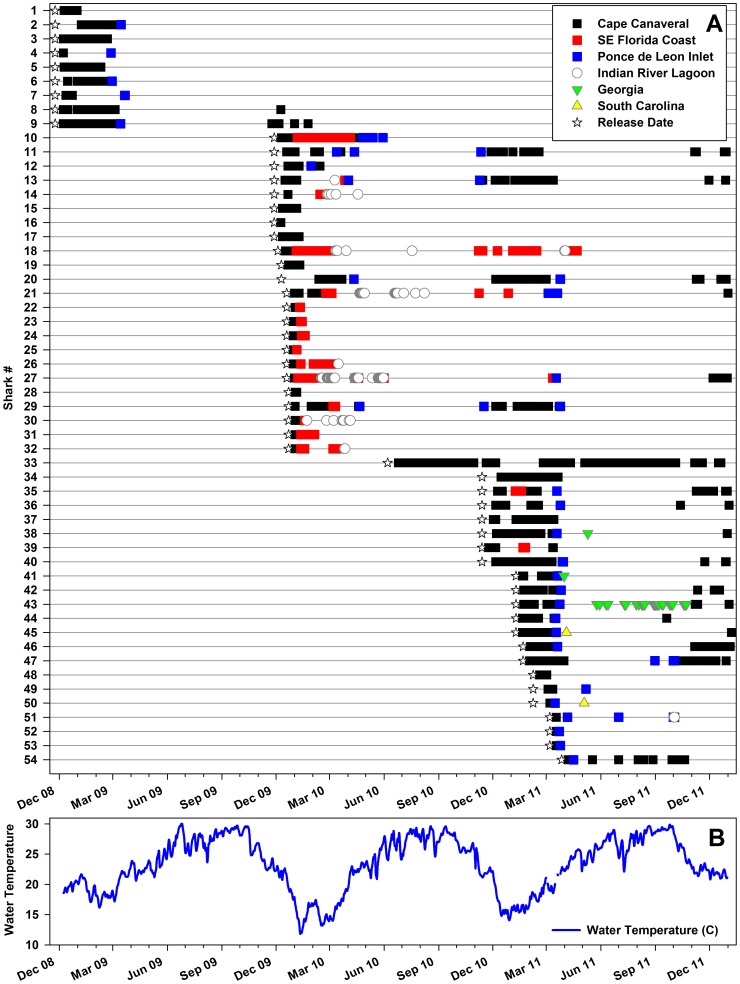
Lemon shark migrations. A) Acoustic detections of all 54 lemon sharks through time, and B) associated nearshore water temperature at Cape Canaveral.

Juvenile lemon sharks were followed for 0.5–751 days with a mean (± 1 SD) of 217 (± 226) days (Table 1; see Table S2 for details on individual sharks). A total of 41,869 position detections were recorded from December 2008 through December 2011 and all 54 sharks were detected in the Canaveral Array at some point. With the exception of early 2010 (see migration details below), tagged sharks generally demonstrated site fidelity to the Cape Canaveral region from late November through late February with few detections elsewhere along the southeastern US coast (Fig. 2). While no shark was detected at Cape Canaveral more than 2600 times, many sharks were recorded here on a near-daily basis for several weeks duration while others were detected more sporadically. The installation of receivers at a second (more northerly) aggregation site in late 2010 demonstrated that individual sharks regularly moved between aggregations and thus commonly spent time beyond the bounds of the initial Canaveral Array footprint.

**Figure 3:**
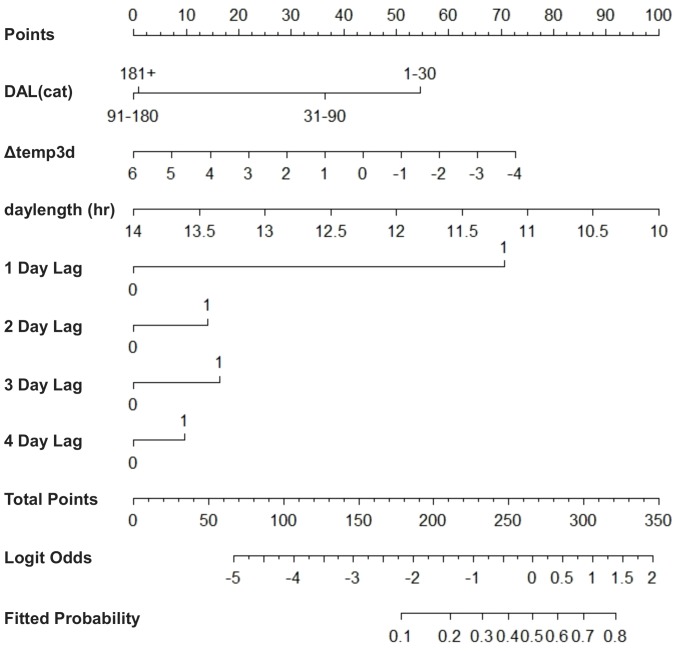
Nomogram depicting effect sizes for the best supported lemon shark residency model. To use the nomogram, locate the desired level of each variable and follow the position vertically up to the Points Scale. Repeat this for all variables and add up the points, then find that value on the Total Points Scale. Finally follow that position directly down to the Fitted Probability Scale which gives the predicted probability of daily detection.

The best-supported residency model (AIC_c_ weight  = 0.87; Table 2) determined that day length, categorical days at liberty, and the magnitude of water temperature change over the previous three days (i.e., Δ3temp) helped predict daily detection probability of lemon sharks at Cape Canaveral. In this model, day length had the greatest (negative) effect size with individuals most likely to be present on the shortest days of the year (Table 3; Fig.3). Δ3temp also had a negative effect meaning that cooling trends resulted in higher predicted probability of shark detection, while warming trends resulted in lower predicted probability. The effect size for days at liberty was also negative meaning that sharks were more often detected on dates nearer their release date. Neither sex nor size helped predict lemon shark presence at Canaveral. Further, an effect of wave height on detection probability, shown during range testing to reduce receiver performance, was not supported, confirming that sharks were detected at least sporadically when present in the Canaveral Array, even during periods of high seas.

**Table 2:** Ten best supported models from the 72 *a priori* models relating environmental and individual covariates to daily detection probability (DDP) of lemon sharks at Cape Canaveral.

Model	k	LogL	Δ AIC_c_^1^	W_i_
DDP ∼ DAL(cat) + Δtemp3d + daylength	12	–2046.5	0.0	0.87
DDP ∼ size + DAL(cat) + daylength	12	–2049.7	6.4	0.03
DDP ∼ DAL(cat) + Δtemp7d + daylength	12	–2049.8	6.6	0.03
DDP ∼ DAL(cat) + daylength + size + sex	13	–2048.8	6.6	0.03
DDP ∼ DAL(cat) + daylength	11	–2052.4	9.8	0.01
DDP ∼ Size(cat) + DAL(cat) + daylength	12	–2051.5	9.9	0.01
DDP ∼ sex + DAL(cat) + daylength	12	–2051.6	10.2	0.01
DDP ∼ DAL(cat) + Δtemp30d + daylength	12	–2051.7	10.5	0.00
DDP ∼ DAL(cat) + daylength + sex + size(cat)	13	–2050.8	10.5	0.00
DDP ∼ DAL(cat) + Δtemp14d + daylength	12	–2052.1	11.1	0.00

All models include state dependence variables (e.g., 1 day lag) to account for any effects of serial autocorrelation, and a random effect for shark and the month by Year. ^1^minimum AIC_c_  = 4117.04.

**Table 3:** Parameter estimates for the best supported state dependence lemon shark residency model. Parameter estimates are also provided for time series modeling for which to compare to state-dependence approach.

	State Dependence				Time Series	
Parameter	Estimate	Std. Error	z value	Pr(>ΙzΙ)	Estimate	Std. Error
Intercept	–3.00	0.23	–12.87	< 2E-16	–2.25	0.22
DAL (cat 31–90)	–0.46	0.16	–2.88	4.00E-03	–0.13	0.13
DAL (cat 91–180)	–1.37	0.22	–6.19	6.20E-10	–1.53	0.21
DAL (cat 181+)	–1.35	0.19	–7.04	1.90E-12	–1.51	0.14
Δtemp3d	–0.16	0.05	–3.37	7.50E-04	–0.15	0.04
daylength	–0.78	0.12	–6.34	2.20E-10	–0.69	0.07
1 day lag	1.77	0.10	17.56	< 2E-16		
2 day lag	0.35	0.12	3.04	2.30E-03		
3 day lag	0.41	0.12	3.46	5.50E-04		
4 day lag	0.24	0.12	2.05	0.04		
**Random Effects**						
shark (SD)	0.94					
year:month (SD)	0.61					

The state dependence variables showed a strong positive correlation between the probability of detection for the 1 day lag, and weaker effects for the 2–4 day lags (Fig. 3). Measures of autocorrelation for Days 1–4 (0.23, 0.05, 0.05, 0.03) agreed well with those estimated by the time series model (0.32, 0.1, 0.03, 0.01). Both methods produced similar parameter estimates, increasing confidence in state dependence modeling for evaluating the effects of covariates on shark detections.

**Figure 4:**
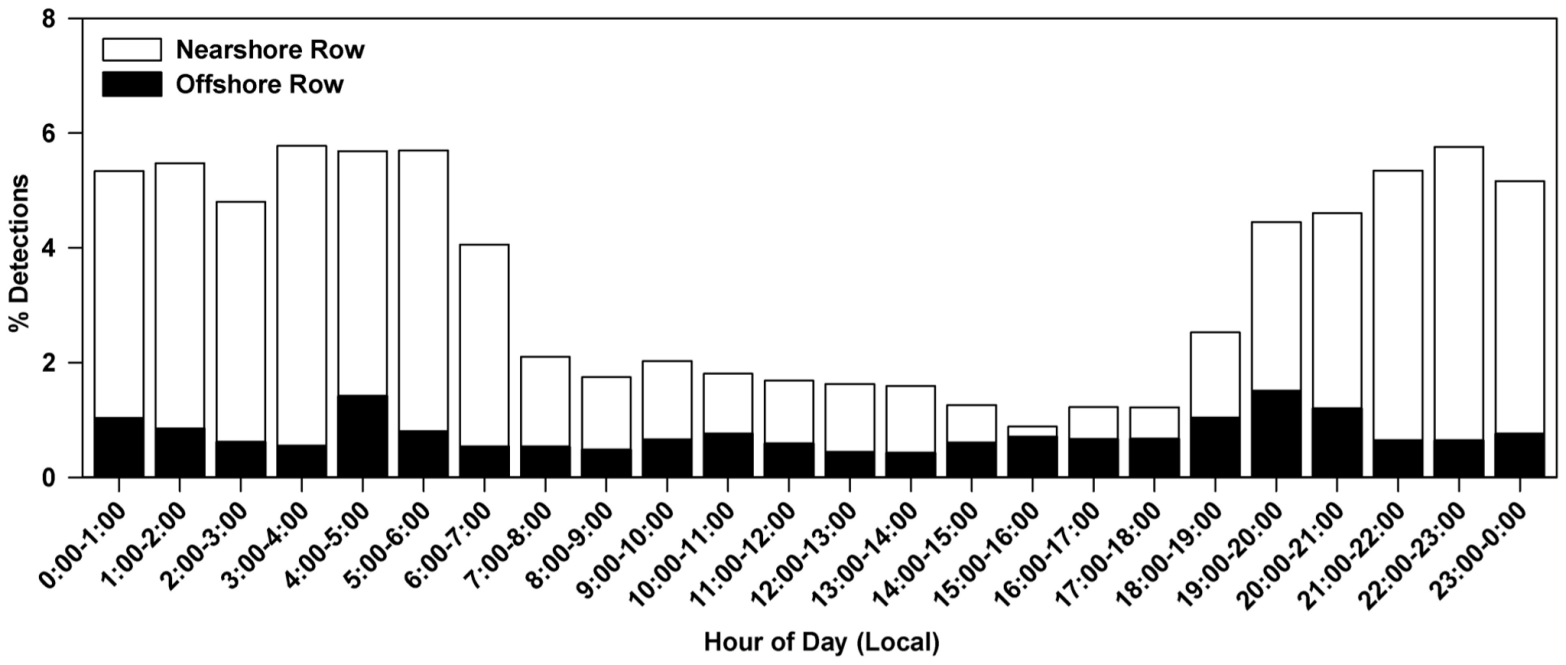
Distribution of lemon shark detections by receiver row and by hour of day . Nearshore receivers were located 250 m from the beach while offshore receivers were 1250 m from the beach.

Lemon sharks were strongly associated with the shoreline when at Cape Canaveral. Nearly 82% of all detections were recorded by the nearshore receiver row, more than expected by chance if sharks used both depths equally (χ^2^ = 9820, df = 1, P<0.001; Fig. 4). Only eight of 42 animals were more commonly detected at offshore receivers, all of which spent little time at Cape Canaveral relative to other sharks. Further, detections were not evenly distributed across the diel period with peak detections occurring at night between 1900–0600 (χ^2^ = 5289, df = 23, *P* <0.001).

### Direction, Timing, and Rate of Coastal Migrations

Of the 54 lemon sharks tagged, 41 were detected away from Cape Canaveral. These individuals were recorded on 62 additional FACT stations from Palm Beach Inlet (26.8° N) to Ponce de Leon Inlet (29.1° N) at various times during the study (Figs. 1,2). Sharks also entered other passive arrays in Ossabaw Sound (n = 3) and Savannah River (n = 1), Georgia (32.0° N), and Charleston Harbor (n = 2), South Carolina (32.8° N). The minimum linear distance between the northernmost and southernmost detection was 663 km but over 770 km when following the coast. On average, sharks were detected on 9.1 receiver stations with individual animals visiting as many as 27 stations. Locational information was also provided via angler recaptures at Jupiter Inlet (170 km south of release site) and Ponce de Leon Inlet, Florida (88 km north of release site), and Little St. Simons Island, Georgia (323 km north of release site).

**Figure 5:**
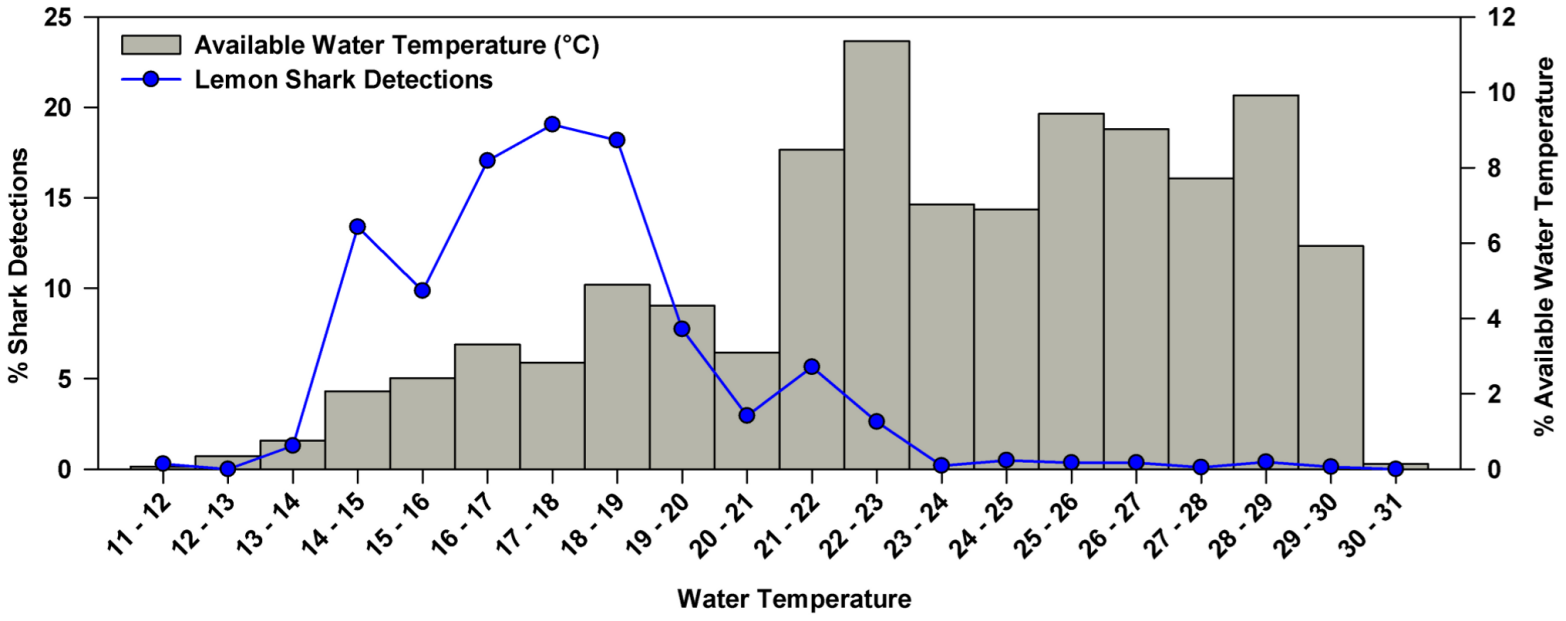
Distribution of available water temperature at Cape Canaveral and associated percentage of lemon shark detections. Water temperature was derived from daily means recorded December 2008 through December 2011.

Nearshore water temperature at Cape Canaveral ranged from 11–30°C and averaged 23.3°C across the study (Fig. 2). Lemon sharks were detected throughout this range (12–30°C) but >70% of detections occurred at temperatures between 15–20°C (Fig. 5).Winter water temperature, averaged from December through March, differed across years (One-Way ANOVA, F = 17.85, *P* <0.001) as a result of severe declines in January-March 2010 and again in December 2010. This atypical variability was accompanied by notable differences in shark migration patterns across the three winters of this study. While extensive records of water temperature are unavailable, local water and air temperatures were strongly correlated (Spearman’s rank correlation, r_s_ = 0.92, df = 982, P<0.001) suggesting that air temperature serves as a good proxy for the relative severity of winters at Cape Canaveral.

The winter of 2008–2009 was moderate with air temperature averaging 17.2°C, (near the long term mean of 17.0 °C) and water temperature ranging from 16–23°C. The nine lemon sharks released in December 2008 were detected locally for 3–106 days with the last two sharks recorded on 5 March (Fig. 2). Five of these sharks were later detected at Ponce de Leon Inlet between 27 February and 22 March 2009 confirming a northward spring migration for these individuals (Table 4). Sharks were not detected elsewhere until sharks #8 and 9 returned to Cape Canaveral on 24 November and 9 December 2009, respectively.

**Table 4:** Date and water temperature (°C) associated with lemon sharks passing by Ponce de Leon Inlet during annual migrations.

Season	No. Sharks	Sex (F:M)	Date Range (Median)	Temperature Range (Median)
Winter-Spring 2009	5	3:2	27 Feb. –22 Mar. (14 Mar.)	17.5–22.0 (19.5)
Fall 2009	0			
Winter-Spring 2010	5	1:4	3 Apr. –26 Apr. (13 Apr.)	17.3–21.6 (20.0)
Fall 2010	3	0:3	9 Nov. –16 Nov. (11 Nov.)	21.0–22.2 (22.1)
Winter-Spring 2011	20	12:8	17 Mar. –7 May (23 Mar.)	16.4–22.3 (16.9)
Fall 2011	2*	1:1	1 Oct. (both sharks)	28.6

Instances where sharks made forays to/past Ponce Inlet but quickly returned to Canaveral (n = 2) are excluded. *Burial of two receivers in fall 2011 limited the ability to detect south-migrating lemon sharks passing by this area.

The 23 lemon sharks released in the second winter of the study were also initially detected only in the Canaveral Array (Fig. 2). By early January 2010, however, several reinforcing cold fronts swept across peninsular Florida resulting in one of the most severe cold weather events on record at Cape Canaveral. Daily air temperature from 2–13 January averaged from 2–10°C, resulting in significant cold-induced mortalities of coastal fishes with tropical affinities (Reyier, personal observation). Moreover, temperatures remained below average for several weeks; winter air temperature averaged only 14.7°C, the sixth coldest on record since 1901. Water temperature in the Canaveral Array reached a minimum of 11°C on 11 January and generally remained below 16°C through mid-March.

This rapid drop in ocean temperature was accompanied by the exodus of all 23 tagged sharks from the Canaveral Array with the last individual (#19) detected on 10 January at a water temperature of 12.0°C. Fifteen sharks made confirmed southward migrations along the coast and were recorded at multiple FACT stations from Sebastian Inlet to West Palm Beach, 62–191 km south of Cape Canaveral. Water temperature in this region was typically 3–6°C warmer than Cape Canaveral due to the moderating influence of the Florida Current which diverges from the Florida east coast near Jupiter. Migrating sharks were always detected singly, a behavior observed consistently across the study. Sharks generally followed the coastline with 13 individuals detected at ocean inlets although one shark was detected 10 km offshore in water 22 m deep. Shark #25 reached West Palm Beach in three days, a rate of 59 km day^−1^ and several others moved at > 40 km day^−1^. Five animals (#22–25, 31) passed by receivers at the south end of FACT at this time and never returned. Other sharks were simply never detected again after leaving the Canaveral Array in early January. Notably, shark #12 actually moved north to Ponce de Leon Inlet in late January (water temperature of 13.6°C) before returning to Cape Canaveral in early February. Eight of 23 tagged sharks returned to the Canaveral Array from 29 January to 8 April. Five of these individuals (#10, 11, 13, 20, 29) were then recorded swimming north past Ponce de Leon Inlet from 3 – 26 April (later in the spring than observed in 2009) with shark #10 subsequently harvested nearby. Like the previous year, the location of these four remaining animals from late spring through fall was undetermined but all four returned to Cape Canaveral between 14 November and 6 December 2010. A single shark (#33) tagged in spring remained within the Canaveral Array throughout the summer 2010 and summer 2011 as well, confirming that at least some juvenile lemon sharks at Cape Canaveral do not undertake northward spring migrations.

December 2010 was also unusually cold; local air temperature averaged 10.8°C, the second coldest December on record since 1901. This event also resulted in mortality of tropical fish species but water temperature was less severe than the previous winter, falling to a low of 14.1°C in late December before returning to more seasonable conditions by early January. Twelve lemon sharks (five released the previous year and seven new releases) were present in the Canaveral Array in early December 2010. While several animals disappeared as water temperature declined, only two (#35, 39) were detected elsewhere in FACT, both near St. Lucie Inlet, 140 km south, and all sharks returned to the Canaveral Array by early January. Further, the 14 animals released later in the winter were never detected south of Cape Canaveral. As in previous years, sharks left the Canaveral Array in spring with 20 individuals recorded at Ponce de Leon Inlet from 17 March to 17 May 2011 (Table 4).

Newly established passive arrays in coastal Georgia and South Carolina provided the first details regarding the destinations and habitat use of north-migrating lemon sharks from spring through fall. Shark #41 was recorded at the mouth of the Savannah River on 31 March. Sharks #45 and 50 were detected within Charleston Harbor on 4 April and 4 May, respectively. Shark #38 was captured and released at Little St. Simon Island, Georgia, on 10 May, and most notably, shark #43 was detected up to 10 km inside Ossabaw Sound, Georgia, on 33 separate dates from May - October 2011, providing a near-complete record of this shark’s location since its release. Despite this extensive northward migration of lemon sharks in spring, 18 of the tagged individuals present at Cape Canaveral during fall-winter of 2010–2011 returned to Cape Canaveral by December 2011 including three of the five sharks (#38, 43, 45) detected in Georgia and South Carolina.

In total, we recorded 72 instances by 40 lemon sharks where individuals travelled > 50 km between receivers. The longest movements (420 km) were observed in two sharks swimming between Ponce de Leon Inlet and Charleston Harbor. Rate of movement ranged from 0.3–63.5 km day^−1^ (mean 18.6 km day^−1^). In cases when migrations occurred within six months of release (i.e., when shark size was known), movement rate measured as body lengths sec^−1^ ranged from 0.02–1.1 (mean 0.27, n = 59). And when considering only migrations along the linear Florida east coast, rates were similar, averaging 18.9 km day^−1^ (n = 66) and 0.27 bl sec^−1^ (n = 53), respectively. Southerly and northerly migrations occurred at similar speeds (t-test, t = –1.384, df = 43.6, *P* = 0.17), were similar across sexes (t-test, t = –0.349, df = 47.1, *P* = 0.72) and were not related to size at capture (Spearman’s rank correlation, r_s_ = 0.02, *P* = 0.88).

### Regional Habitat Use

Tagged lemon sharks were detected within every major habitat monitored by the FACT Array. While all 54 sharks (73% of all detections) were recorded in nearshore Atlantic waters, 40 sharks (19% of detections) were also recorded at tidal inlets including Ponce de Leon (n = 30), Sebastian (n = 2), Ft. Pierce (n = 9), St. Lucie (n = 7), and Jupiter Inlets (n = 6) as well as nearby Port Canaveral (n = 2). Nine animals (7% of detections) penetrated >5 km into estuarine waters of the Indian River Lagoon and one shark (1% of detections) was recorded 7 km up the Loxahatchee River near Jupiter Inlet although salinity at this site was not available. Shark #21 spent ≥ 166 days in the estuary and moved 106 km north from Sebastian Inlet before returning south and offshore, spending more time and moving farther up-estuary than any other tagged individual. With the exception of Ponce de Leon Inlet, which lies along the annual migration route, use of inlet and estuarine habitats within the FACT Array occurred almost exclusively during early 2010 as sharks moved south from Cape Canaveral in association with rapidly falling water temperature.

## Discussion

In this study, we utilized a collaborative passive acoustic array to document regional-scale migrations and habitat associations of juvenile lemon sharks in the US South Atlantic for the first time. Tagged sharks utilized at least 660 km of coastline from southeast Florida to South Carolina with individuals tracked for up to 751 days. Our findings clearly demonstrated that: (1) immature lemon sharks found in nearshore aggregations at Cape Canaveral exhibited site fidelity to this region from December through February under seasonally typical water temperatures; (2) temperature declines below 15°C were accompanied by a rapid but often temporary southward displacement along the Florida east coast; and (3) in contrast to other populations studied to date, most juvenile lemon sharks overwintering in east-central Florida undertook an annual northward migration starting in late winter, and spent summer in nearshore and estuarine waters of north Florida, Georgia, and the Carolinas before returning south to east-central Florida in fall.

### Cape Canaveral as a Lemon Shark Nursery

The notion that many coastal shark species have discrete nurseries has been widely accepted for decades with many adopting the definition of Bass [53] who states that primary nurseries are locations where parturition takes place and secondary nurseries are where young reside when growing to maturity. Huepel et al. [54] argue convincingly that this concept is too often applied to areas where immature sharks occur in low density or spend little time. They instead propose three testable criteria for evaluating whether a location is indeed a shark nursery: (1) young sharks of a given species are more abundant than in other areas, (2) individuals use the putative nursery for extended periods (i.e., exhibit site fidelity), and (3) the area is utilized by a species repeatedly across years.

Our growing understanding of lemon shark life history in the US South Atlantic suggests that nearshore waters at Cape Canaveral merit the definition of a winter nursery for the species even under these stricter standards, and may constitute the single most valuable winter nursery for lemon sharks in US waters north of the Florida Keys-Florida Bay region. While abundance was not quantified here, tagged sharks were sampled from aggregations of several hundred individuals, and winter densities as high as 22 sharks per shoreline km have been observed locally in recent years [42]. To our knowledge, this aggregating behavior has not been noted for juveniles elsewhere along the US Atlantic coast, and immature lemon sharks are a minor component of shark surveys elsewhere in Florida [29], [55], Georgia [56], South Carolina [57], [58], and North Carolina [59]. Our findings directly address more challenging questions regarding site fidelity and seasonal philopatry (i.e., homing) to the Canaveral region. The FACT Array provided strong evidence that most juvenile lemon sharks arrived at Cape Canaveral beginning in late November, remained through February (often longer), and utilized coastal waters south of Cape Canaveral only when water temperature receded below 15°C. And while aggregations dissipated each spring, they reformed the ensuing winter, as they have annually since first encountered in 2003 [42]. Most notably, 19 of 54 tagged individuals returned for a second or even third successive winter. Given that mortality of young lemon sharks has been estimated at 38–65% annually [60], and that transmitters deployed the first winter had battery life < 1 year, this rate of return appears high.

The reason(s) why lemon sharks aggregate at Cape Canaveral is not fully understood but our data suggest that water temperature largely underlies this phenomenon. Cape Canaveral is a climatic transition zone where winter water temperature grades rapidly from north to south and does not drop below 15 °C most years [45]. This condition is partially a function of latitude, however satellite ocean temperature imagery also suggests that the nearby shoal complex partially deflects the predominant south-flowing nearshore current eastward, allowing warmer north-flowing offshore currents to intrude near the coast. On some winter days, water temperature on either side of the shoals may differ by up to 2–3°C. In most years, therefore, the Canaveral region may simply be the highest latitude where lemon sharks can safely overwinter without serious repercussions to survival and growth. Since tagged sharks returned to Canaveral as early as November when water temperature in northeast Florida was still typically >20°C, aggregations may be an instinctive or learned behavior, not a direct response to ambient temperatures encountered during southward fall migrations. The sand shoals here may also serve as a predator refuge or productive foraging grounds. In fact, following the conclusion of this study, the Canaveral Array was further expanded with receivers deployed further offshore. To date, a total of 13 juvenile lemon sharks have been detected up to 12 km from the beach (E. Reyier, unpubl. data). Finally, it is conceivable that these juvenile aggregations were historically more widespread in east Florida during winter but now persist only at Cape Canaveral due to limits on public shore access and fishing enacted for space launch security in the 1950s.

### Seasonal Migrations in the US South Atlantic

The historically cold water temperature during January 2010 resulted in widespread mortality of tropical fish species throughout peninsular Florida [61], but was fortuitous in the sense that it allowed us to observe a broader suite of lemon shark behavior than might be expected in a typical three year period. Like other marine fishes, lemon sharks exposed to temperature approaching their lower lethal limit would be subject to disruption of neuroendocrine, metabolic, osmoregulatory, and immune functions, potentially culminating in death [62]. The sudden exodus of all tagged lemon sharks from Cape Canaveral once water reached 12°C in early 2010, and a rapid southern migration of at least 15 individuals to coastal waters moderated by the warm Florida Current, was clearly in direct response to this unusual meteorological event. The near-complete exodus of sharks from Cape Canaveral from February through April in all three years of the study and the subsequent detections of 30 individuals at Ponce de Leon Inlet (northeast Florida), Georgia, and South Carolina, demonstrate that lemon sharks as small as 660 mm FL commonly undertake extensive northward migrations each spring. In contrast to southern migrations observed in early 2010, these annual migrations may not be cued directly by water temperature. Day length, not temperature, appeared as single most important factor when predicting lemon shark presence at Cape Canaveral over the long term, and many north-migrating sharks passed Ponce de Leon Inlet when water temperature was only 16–18°C. We suggest that growing day length in spring provides the primary stimulus to initiate annual coastal migrations, as has also been been suggested for sandbar sharks (*Carcharhinus plumbeus*) in Chesapeake Bay [11].

The extensive migrations we observed contrast with results of virtually every other study of lemon shark behavior and dispersal in the Bahamas [33], [35], Caribbean [39], Brazil [24], and even south Florida [38]. Most notably, Chapman et al. [34] used genetic techniques to conclude that dispersal of lemon sharks in Bimini, Bahamas (only 320 km from Cape Canaveral), was very slow; the majority of individuals up to six years old at Bimini were locally born. Most previous studies have occurred at insular sites or lower latitudes where seasonal migrations may be less advantageous because annual temperature variability is less extreme, or because dispersal is not attempted - or not often successful - due to high juvenile mortality in the open ocean. Regular lemon shark migrations along the US southeast coast are presumably an adaption which allows seasonal use of productive estuaries from spring through early fall as temperatures allow. These migrations also result in lower densities which may be necessary since the condition of lemon sharks in aggregations deteriorates as winter progresses [42], suggesting that Canaveral waters cannot sustain such high shark numbers year-round.

The stark regional differences in lemon shark behavior and habitat associations underscore the wisdom of tailoring management strategies to both a species basic biology, which may vary little over broad geographic scales, and its behavior, which varies from site to site. Along the US east coast, lemon sharks are currently managed as a single stock in the large coastal shark management group [63], subject to recreational and commercial size and catch quotas. Further, In 2010, due to mounting evidence that lemon shark aggregating behavior made them especially vulnerable to overfishing, the State of Florida imposed an outright, although potentially temporary, harvest ban in state waters [43]. Given the extensive migrations we observed in individual sharks, coupled with the spatially predictable nature of their aggregations, this dual approach seems warranted. That said, permanent protection of Florida’s lemon shark aggregations in both state and federal waters (possibly through extremely stringent quotas or time-area closures) may be the single most important step for ensuring long-term conservation of the species in the US south Atlantic region.

### Remaining Questions

Adult lemon sharks that overwinter off Jupiter, Florida, exhibit a similar north-south migratory pattern along the coast. Almost 60 tagged adults passed through the Canaveral Array in late spring and several remained in the region well into summer. Female lemon sharks give birth in spring but the apparent lack of neonates at Cape Canaveral or adjacent estuary [42] suggests that parturition occurs primarily north of east-central Florida; to date these pupping areas have not been located. And while ongoing genetic sampling has demonstrated that adults in Jupiter aggregations are the parents of some Canaveral juveniles (D. Chapman, unpubl. data), it remains unclear to what extent, and at what age, the immature sharks recruit into adult aggregations down the Florida coast. That said, this study validates the use of collaborative passive arrays for the purposes of resolving regional-scale migrations for managed coastal fishes not easily tracked in detail with satellite-based techniques. As the technology becomes more widely embraced, answers to these questions will be within reach for lemon sharks and other coastal shark species.

## Supporting Information

Table S1 Canaveral Array Performance. The best supported generalized least squares model for the receiver performance trial had main effects for wave height and temperature. Test distance between transmitter and receivers was 500 m.
(DOCX)

Table S2 Detailed information for all 54 lemon sharks tagged at Cape Canaveral. Days at Liberty equals the number of days between date of release and date of last detection within the acoustic array. Maximum displacement means the farthest known detection (in km) north and south of release point. Asterisk indicates angler capture. Stations visited includes all FACT and non-FACT locations.
(DOCX)
