# Supplementary material for: Regional-Scale Migrations and Habitat Use of Juvenile Lemon Sharks (Negaprion brevirostris) in the US South Atlantic
Source: PLoS One. 2014 Feb 26;9(2):e88470. doi: 10.1371/journal.pone.0088470 (PMC3935833; doi:10.1371/journal.pone.0088470)
Supplement: Table S1 — Canaveral Array Performance. The best supported generalized least squares model for the receiver performance trial had main effects for wave height and temperature. Test distance between transmitter and receivers was 500 m. (DOCX) [file pone.0088470.s001.docx]

| **Table S1. Canaveral Array Performance.** The best supported generalized least squares model for the receiver performance trial had main effects for wave height and temperature. Test distance between transmitter and receivers was 500 m. | | | | |
| --- | --- | --- | --- | --- |
| **Variable** | **Estimate** | **SE** | **t value** | **Pr(>\|t\|)** |
| Wave Height | -15.31 | 1.25 | -12.27 | < 0.001 |
| Temperature | 11.08 | 1.25 | 8.88 | < 0.001 |
| Intercept | 63.34 | 1.25 | 51.15 | < 0.001 |
|  |  |  |  |  |
| Parameter estimates shown are for the standardized variables | | | |  |
