# Supplementary material for: Regional-Scale Migrations and Habitat Use of Juvenile Lemon Sharks (Negaprion brevirostris) in the US South Atlantic
Source: PLoS One. 2014 Feb 26;9(2):e88470. doi: 10.1371/journal.pone.0088470 (PMC3935833; doi:10.1371/journal.pone.0088470)
Supplement: Table S2 — Detailed information for all 54 lemon sharks tagged at Cape Canaveral. Days at Liberty equals the number of days between date of release and date of last detection within the acoustic array. Maximum displacement means the farthest known detection (in km) north and south of release point. Asterisk indicates angler capture. Stations visited includes all FACT and non-FACT locations. (DOCX) [file pone.0088470.s002.docx]

| **Table S2. Detailed information for all 54 lemon sharks tagged at Cape Canaveral.** | | | | | | | | |
| --- | --- | --- | --- | --- | --- | --- | --- | --- |
|  |  |  |  |  |  |  |  |  |
| **Shark** | **Release Date** | **Fork Length (mm)** | **Sex** | **Days at Liberty** | **Detects** | **Maximum Displacement (N/S)** | **Tag Code** | **Stations Visited** |
| 1 | 12/05/08 | 645 | M | 26 | 373 | 3/2 | 9598 | 4 |
| 2 | 12/05/08 | 870 | F | 101 | 1095 | 0/84 | 9599 | 4 |
| 3 | 12/05/08 | 890 | F | 78 | 577 | 3/2 | 9600 | 4 |
| 4 | 12/05/08 | 900 | F | 84 | 42 | 0/84 | 9601 | 2 |
| 5 | 12/05/08 | 810 | M | 66 | 111 | 3/0 | 9602 | 3 |
| 6 | 12/05/08 | 870 | M | 86 | 293 | 2/84 | 9603 | 4 |
| 7 | 12/05/08 | 690 | F | 107 | 26 | 3/84 | 9604 | 6 |
| 8 | 12/05/08 | 1050 | M | 370 | 564 | 2/2 | 9605 | 4 |
| 9 | 12/05/08 | 1080 | F | 416 | 2279 | 2/84 | 10008 | 5 |
| 10 | 12/09/09 | 1210 | M | 172* | 327 | 172/91 | 63219 | 17 |
| 11 | 12/09/09 | 940 | M | 751 | 717 | 3/84 | 63220 | 13 |
| 12 | 12/09/09 | 740 | M | 66 | 1699 | 3/84 | 63221 | 9 |
| 13 | 12/09/09 | 1080 | M | 749 | 1292 | 152/69 | 63222 | 23 |
| 14 | 12/09/09 | 890 | F | 130 | 123 | 183/0 | 63223 | 11 |
| 15 | 12/09/09 | 610 | M | 28 | 784 | 3/4 | 63224 | 6 |
| 16 | 12/09/09 | 680 | F | 0.5 | 16 | 1/3 | 63225 | 2 |
| 17 | 12/09/09 | 710 | M | 31 | 1651 | 2/4 | 63226 | 7 |
| 18 | 12/15/09 | 675 | M | 493 | 752 | 169/0 | 63230 | 19 |
| 19 | 12/21/09 | 715 | F | 21 | 1372 | 3/2 | 63218 | 7 |
| 20 | 12/21/09 | 1008 | F | 738 | 1212 | 6/80 | 63231 | 13 |
| 21 | 12/30/09 | 695 | M | 732 | 1458 | 110/83 | 63227 | 27 |
| 22 | 12/30/09 | 686 | M | 12 | 394 | 172/0 | 63228 | 8 |
| 23 | 12/30/09 | 615 | M | 15 | 270 | 166/2 | 63229 | 9 |
| 24 | 12/30/09 | 705 | F | 20 | 66 | 173/0 | 63232 | 6 |
| 25 | 12/30/09 | 655 | F | 6 | 43 | 191/3 | 63233 | 5 |
| 26 | 12/30/09 | 688 | M | 77 | 599 | 146/4 | 63234 | 13 |
| 27 | 12/30/09 | 730 | F | 730 | 9080 | 171/85 | 63235 | 13 |
| Days at Liberty equals the number of days between date of release and date of last detection within the acoustic array. Maximum displacement means the farthest known detection (in km) north and south of release point. Asterisk indicates angler capture. Stations visited includes all FACT and non-FACT locations. | | | | | | | | |
|  |  |  |  |  |  |  |  |  |
|  |  |  |  |  |  |  |  |  |

|  | |  |  |  |  |  |  |  |
| --- | --- | --- | --- | --- | --- | --- | --- | --- |
| **Shark** | **Release Date** | **Fork Length (mm)** | **Sex** | **Days at Liberty** | **Detects** | **Maximum Displacement (N/S)** | **Tag Code** | **Stations Visited** |
| 28 | 01/02/10 | 680 | F | 3 | 136 | 4/0 | 62969 | 3 |
| 29 | 01/02/10 | 1004 | M | 447 | 527 | 116/83 | 62970 | 17 |
| 30 | 01/02/10 | 715 | F | 93* | 446 | 172/1 | 62971 | 10 |
| 31 | 01/02/10 | 728 | F | 33 | 506 | 172/1 | 62972 | 13 |
| 32 | 01/02/10 | 695 | F | 84 | 43 | 167/2 | 62973 | 8 |
| 33 | 06/18/10 | 1430 | F | 551 | 666 | 23/6 | 62971-2 | 11 |
| 34 | 11/24/10 | 720 | M | 117 | 931 | 20/8 | 45102 | 14 |
| 35 | 11/24/10 | 908 | M | 401 | 214 | 155/68 | 45103 | 12 |
| 36 | 11/24/10 | 875 | M | 323 | 206 | 13/69 | 45104 | 8 |
| 37 | 11/24/10 | 1172 | M | 110 | 365 | 14/10 | 45105 | 5 |
| 38 | 11/24/10 | 930 | M | 403* | 338 | 19/311 | 45107 | 11 |
| 39 | 11/24/10 | 674 | M | 109 | 797 | 154/0 | 45109 | 14 |
| 40 | 11/24/10 | 1013 | F | 401 | 456 | 21/64 | 45111 | 13 |
| 41 | 01/20/11 | 770 | F | 70 | 226 | 3/403 | 45110 | 13 |
| 42 | 01/20/11 | 950 | F | 333 | 556 | 17/66 | 45113 | 9 |
| 43 | 01/20/11 | 1230 | M | 295 | 621 | 16/366 | 45115 | 10 |
| 44 | 01/20/11 | 885 | M | 244 | 318 | 18/69 | 45117 | 14 |
| 45 | 01/20/11 | 793 | F | 345 | 1510 | 18/472 | 45119 | 14 |
| 46 | 02/01/11 | 660 | F | 331 | 2410 | 3/84 | 45106 | 11 |
| 47 | 02/01/11 | 695 | M | 331 | 2641 | 18/69 | 45108 | 12 |
| 48 | 02/18/11 | 1110 | M | 12 | 369 | 2/27 | 45114 | 9 |
| 49 | 02/18/11 | 739 | M | 78 | 228 | 1/68 | 45116 | 4 |
| 50 | 02/18/11 | 695 | F | 75 | 13 | 2/470 | 45120 | 4 |
| 51 | 03/18/11 | 732 | F | 199 | 74 | 0/69 | 45101 | 4 |
| 52 | 03/18/11 | 940 | F | 5 | 4 | 0/69 | 45112 | 2 |
| 53 | 03/18/11 | 680 | F | 7 | 8 | 0/69 | 45118 | 1 |
| 54 | 04/07/11 | 1121 | F | 196 | 45 | 8/61 | 63219-2 | 4 |
| **Mean** |  | **840** |  | **216** | **775** | **56/74** |  | **9.1** |
